# Combination of Modafinil and *d*-amphetamine for the Treatment of Cocaine Dependence: A Preliminary Investigation

**DOI:** 10.3389/fpsyt.2012.00077

**Published:** 2012-08-30

**Authors:** Joy M. Schmitz, Nuvan Rathnayaka, Charles E. Green, F. Gerard Moeller, Anne E. Dougherty, John Grabowski

**Affiliations:** ^1^Department of Psychiatry and Behavioral Sciences, University of TexasHouston, TX, USA; ^2^Center for Clinical Research and Evidence-Based Medicine, University of TexasHouston, TX, USA; ^3^Department of Internal Medicine (Cardiology), University of TexasHouston, TX, USA; ^4^Department of Psychiatry, University of MinnesotaTwin Cities, MN, USA

**Keywords:** cocaine dependence, clinical trial, modafinil, *d*-amphetamine

## Abstract

**Background:** Two stimulant medications, modafinil and *d*-amphetamine, when tested individually, have shown safety and efficacy for treatment of cocaine addiction. We hypothesized that the combination of modafinil and *d*-amphetamine, at low doses, would show equivalent or greater benefit in reducing cocaine use compared to higher doses of each individual medication or placebo. **Methods:** Sixteen week, randomized, parallel-group design with four treatment arms comparing placebo to modafinil 400 mg; *d*-amphetamine 60 mg; modafinil 200 mg plus *d*-amphetamine 30 mg. Primary outcome variables, retention and cocaine use, were analyzed on the sample of 73 participants who received the first dose of the study medication. **Results:** Retention rates did not differ between groups and were generally low, with 40% remaining in treatment at week 12 and 20% at week 16. Participants receiving the combination of modafinil and *d*-amphetamine showed a trend of increased cocaine use over time with a corresponding low Bayesian probability of benefit (33%). Relatively better cocaine outcomes were observed in the placebo and *d*-amphetamine only groups. The study medications were generally well-tolerated with few adverse effects, yet rates of adherence were suboptimal (≤80%). **Conclusion:** Data from this preliminary investigation fail to provide evidential support for conducting a larger study of this dual-agonist medication combination for treatment of cocaine dependence.

## Introduction

A number of candidate stimulant-like agents have been studied for the treatment of cocaine dependence based on the “agonist substitution” model, similar to the use of methadone to treat opiate dependence. By definition, an agonist-like medication should possess neurochemical and behavioral effects similar to those of the abused drug, with diminished abuse liability (Herin et al., 2010). Given the key role of dopamine in the acute and chronic effects of cocaine, drugs that interact with this monoamine neurotransmitter have been prime targets for medication development research (Moeller et al., 2008). Medications enhancing dopaminergic function that have been examined with promising results include, e.g., bupropion, disulfiram, methylphenidate, levodopa, methamphetamine (Margolin et al., 1995; Grabowski et al., 1997; Petrakis et al., 2000; Carroll et al., 2004; Poling et al., 2006; Schmitz et al., 2008; Mooney et al., 2009).

Two stimulant medications, modafinil and *d*-amphetamine, when tested individually, have shown some evidence of safety and benefit for cocaine addiction. Consistent with its ability to weakly inhibit DA reuptake, the behavioral effects of modafinil overlap to some extent with those of prototypical stimulants, including enhanced extracellular DA in the nucleus accumbens (Ferraro et al., 1996; Madras et al., 2006). In two early clinical trials, cocaine dependent subjects receiving modafinil were more likely to submit cocaine-negative urine samples than subjects receiving placebo (Dackis et al., 2005). More recently, Anderson et al. (2009) found a significant effect of modafinil that increased weekly percentage of cocaine non-use days in the subgroup of cocaine dependent patients without comorbid alcohol dependence, but not in the total sample. *d*-Amphetamine enhances dopaminergic functioning via reversal of the dopamine transporter, and has been tested in three placebo-controlled trials. Two studies by the same group (Grabowski et al., 2001, 2004) reported a reduction in cocaine-positive urine drug screens in cocaine dependent individuals treated with sustained-release *d*-amphetamine up to 60 mg/day compared with placebo-treated individuals. One of these studies used dual-diagnosis cocaine-opiate – dependent individuals also being treated with methadone (Grabowski et al., 2004). Shearer et al. (2003) conducted a controlled community trial with a heterogeneous population of cocaine users in which oral immediate-release *d*-amphetamine was administered. Whereas outcomes favored the treatment group in terms of cocaine use measured by urinalysis and self-report, improvements were not statistically significant in this small sample (*N* = 30) pilot study. Longo et al. (2010) reported *d*-amphetamine produced enhanced retention and decreased illicit use in a methamphetamine abusing population. These reports, along with multiple community clinic and case series reports of reductions in amphetamine abuse during *d*-amphetamine maintenance, point to potential utility.

Thus, agonist-like medications for cocaine and methamphetamine dependence have been examined in several studies though the results are not consistent and further large randomized trials are required. New strategies have been recommended, including use of agonist combinations (Shearer, 2008; Herin et al., 2010). The use of modafinil with *d*-amphetamine as a treatment strategy is based on the rationale that the combination should more adequately provide “substitution” or “replacement” of depleted brain monoamines. Each agent enhances dopaminergic transmission, though via different mechanisms, potentially resulting in broader actions that target also the norepinephrine, glutamate, and other systems modulated by cocaine. Given the documented safety and tolerability of each agent at low doses, the likelihood of side effect or adverse events is low and offset by the likelihood of incremental benefit expected from the combination. For this preliminary investigation, we tested the hypothesis that the low dose combination of modafinil (200 mg/day) and *d*-amphetamine (30 mg/day) would produce greater benefit in reducing cocaine use compared to higher doses of each individual medication or placebo.

## Methods

### Study design

This double-blind, placebo-controlled randomized clinical trial compared 400 mg of modafinil to 60 mg of sustained-release *d*-amphetamine to the combination of 200 mg modafinil and 30 mg of *d*-amphetamine in a sample of 73 subjects with cocaine dependence. Following assignment to one of the four treatment groups, subjects attended thrice weekly clinic visits, weekly individual therapy, and received study medication for 16 weeks. Following a 2-week intake evaluation phase, baseline information was used to urn randomize subjects to ensure balanced groups with respect to gender and severity of cocaine addiction (Stout et al., 1994). Treatment began with a 1-week dose escalation schedule, described below, followed by maintenance for 16 weeks, and a 1-week dose reduction at week 17.

### Subjects

All participants were enrolled at the outpatient Treatment Research Clinic (TRC) located at the Center for Neurobehavioral Research on Addictions in Houston, Texas. Inclusion required being between 18 and 55 years old and meeting Diagnostic and Statistical Manual of Mental Disorders-IV (DSM-IV) criteria for current cocaine dependence with provision of at least one benzoylecgonine (BE)-positive urine during the intake evaluation period prior to randomization. Exclusion criteria included: (1) dependence on alcohol or drugs other than cannabis or nicotine; (2) current non-substance induced Axis I psychotic, depressive, or anxiety disorder; (3) presence of existing cardiovascular disease as determined by EKG evaluated by the collaborating cardiologist (AED), and/or symptoms suggestive of cardiovascular problems not related to drug use such as hypertension (treated or untreated), stroke, chest pain; (4) taking medications that would contraindicate study medications (e.g., MAO inhibitors, tricyclic antidepressants, SSRI’s); (5) pregnancy or nursing; and (6) court-mandated treatment for cocaine dependence.

The TRC conducts an initial eligibility screening on all individuals who call in response to ads for various treatment studies (Sayre et al., 2004). Those who qualify are further screened for one of several ongoing clinical trials. Of the 84 subjects who were determined as being eligible to participate in the current trial, 11 did not return for their study start visit and were thus lost to follow-up, leaving a total of 73 subjects in the randomized, intent-to-treat, sample.

The research protocol, consent form, and all assessment/advertising materials were reviewed and approved by the Committee for the Protection of Human Subjects (CPHS) of the University of Texas Medical School, Houston (Clinicaltrials.gov Identifier: NCT00218062).

### Assessments

Psychiatric diagnostic and addiction severity information were collected at intake using the Structured Clinical Interview for DSM-IV (Spitzer and First, 2005) and the Addiction Severity Index (McLellan et al., 1992). Prior to starting medication, all subjects underwent a medical history and physical examination, laboratory tests (liver and thyroid function), and cardiac evaluation (i.e., 12-lead electrocardiogram). Vital signs (including heart rate, blood pressure, and weight) were obtained weekly during treatment. EKG’s were conducted at the time of screening and repeated every 4 weeks during treatment. Blood pressure was closely monitored throughout the study, with a requirement of being normotensive (<140/90) to receive study medications. A side-effects checklist was completed each week, with moderate to severely rated items evaluated by the study nurse and reviewed by the study physician (FGM). Medication compliance was assessed via fluorescent tests for riboflavin detection in urine samples (see below) and by self-reported number of pills taken each day, as measured using a written Timeline Followback (TLFB) method. Cocaine use was assessed via urinalysis results detecting the presence of BE (≥300 mg/ml) and by self-reported cocaine use as recorded on the TLFB.

### Treatments

During the 16-weeks of outpatient treatment, participants took three capsules daily (two in the morning, one in the afternoon). All active and placebo capsules were identical in appearance and each contained 50 mg riboflavin for subsequent evaluation of medication compliance (Del Boca et al., 1996). Medication administration was initiated during a 5-day run-up period. Modafinil started at 200 mg (day 1) and increased to 400 mg (days 2–5). *d*-Amphetamine SR (Dexedrine Spansules) started at 15 mg (day 1–2), increased to 30 mg (day 3; 15 mg, BID), 45 mg (day 4; 15 mg, TID), and 60 mg (day 5; 15 mg bid plus 30 mg qd). For the combination condition, dosages of modafinil and *d*-amphetamine were escalated to one-half of that for the single medication conditions. A 5-day dose reduction schedule occurred at week 17. All investigators and staff, except the pharmacist, were blind to medication assignment.

Manual-based, cognitive-behavioral therapy was provided for 1 h each week by master’s-level therapists. The cognitive-behavioral therapy emphasized relapse prevention and coping skills (for a full description see Schmitz et al., 2001).

### Data analytic plan

Primary outcome variables, retention, and cocaine use, were analyzed on the sample of participants who were randomized to treatment and received the first dose of the study medication. Demographic and baseline characteristics of study participants by group were compared using analysis of variance (ANOVA) and Fisher’s exact test for continuous and categorical variables, respectively. For retention, group differences in the percentage of subjects remaining in treatment over the 16-week study were tested using Kaplan–Meier survival analysis. The primary outcome of cocaine use was examined using urine toxicology results. Treatment differences in weekly fraction of cocaine-positive urines were compared using generalized linear mixed models (GLMM) for repeated measures analysis including terms for treatment, time, and treatment by time interaction. The primary null hypothesis, that there was no difference between medication groups in primary outcome measures, was tested using the traditional Frequentist approach at a 5% level of statistical significance. Significant interactions were followed by *post hoc* tests of the simple effects of time within each treatment to determine if differences were driven by greater change in the combination group relative to other treatment groups. We then applied exploratory Bayesian analyses to add interpretive statements regarding the probability that the alternative hypothesis exists, i.e., that treatment conferred benefit given the observed data, expressed as the probability that the odds ratio for each simple effect was less than 1.0.

Whereas the classical frequentist approach constructs a rejection region and reports the probability that the null hypothesis obtains, based on an all or nothing dichotomy (*p* ≤ 0.05), the Bayesian approach constructs prior and posterior distributions to *quantify* the probability of each hypothesis (null and alternative), given observed data. Together, these two complementary approaches can provide a more accurate parameter estimate when the sample size is small (for a full description, see Bayarri and Berger, 2004; Green et al., 2009).

## Results

### Sample characteristics

The demographic and substance use characteristics of participants at randomization are presented in Table 1. The mostly male (85%), African American (64%) sample had a mean age of 42 years (SD = 8.1), a mean education level of 13 years (SD = 1.7), and a reported unemployment rate of 60%. Recent cocaine use was reported to be 17.9 (SD = 8.5) days in the past 30, with lifetime cocaine use reported to be 13.6 (SD = 7.7) years. The treatment groups were not significantly different on these baseline characteristics.

**Table 1:** **Demographic and drug use characteristics of participants at baseline by randomization status**.

Variable	*d*-Amphetamine *n* = 22		Modafinil *n* = 20		Modafinil and *d*-amphetamine *n* = 15		Placebo *n* = 16	
	*N*	%	*N*	%	*N*	%	*N*	%
Male	21	95.5	16	84.2	11	73.3	14	87.5
White	6	27.3	6	31.6	3	20.0	2	12.5
Black	15	68.2	12	63.2	10	66.7	10	62.5
Hispanic	1	4.6	1	5.3	2	13.3	3	18.8
Employment (unemployed)	16	72.7	13	68.4	10	71.4	5	31.3

	**Mean**	**SD**	**Mean**	**SD**	**Mean**	**SD**	**Mean**	**SD**

Age	44.3	6.5	42.6	8.3	41.2	8.5	41.9	9.0
Education (years)	12.7	1.6	12.9	1.5	13.2	2.0	13.5	1.8
Cocaine use (past 30 days)	17.1	8.8	16.4	8.6	20.5	7.3	17.7	9.6
Lifetime cocaine (years)	12.9	8.1	12.4	7.5	13.9	8.0	15.5	7.4
Alcohol use (past 30 days)	10.6	10.8	10.8	9.9	6.4	9.0	10.1	8.5
Lifetime alcohol (years)	20.5	11.3	17.8	11.4	16.5	13.9	19.5	12.9

### Retention in treatment

Rates of retention during treatment did not differ between groups, log rank χ^2^ (1) = 1.307, *p* = 0.72, and were generally low, with 40% remaining in treatment at week 12 and 20% at week 16. Overall median number of days in treatment was 52 (95% C.I. 28–84).

### Cocaine use

**Figure 1:**
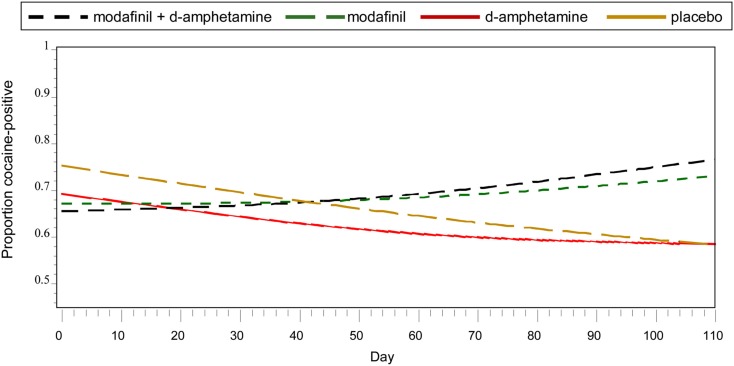
**Probability of cocaine use by medication condition and time**.

Frequentist analyses modeled cocaine-positive urines using linear and quadratic temporal trends, the main effects of treatment, and the interaction of the linear trend and treatment (Figure 1). A statistically reliable time by treatment interaction, *F* (3, 921) = 2.93, *p* = 0.03, indicated differential change over time as a function of treatment condition. Simple effects of time within each treatment condition suggested that, for the placebo group, the odds of having a cocaine-positive urine decreased significantly for every additional day in treatment (OR = 0.980, 95% CI 0.973–0.987). As shown in Table 2, Bayesian estimates produced similar results, while offering the alternative interpretation of there being a 98.5% chance that placebo conferred benefit (i.e., OR <1) in reducing the probability of cocaine-positive urines, given the current data. A similar trend of decreased cocaine use over time with a corresponding high Bayesian probability of benefit (77.2%) was found in the *d*-amphetamine only group. For the conditions of modafinil + *d*-amphetamine and modafinil only, the non-significant simple effects of time suggested increased cocaine use and were supported by corresponding low Bayesian probabilities of benefit (i.e., OR’s <1), 14.0 and 33.0%, respectively.

**Table 2:** **Frequentist and Bayesian results for the simple effects of time within each treatment condition on cocaine-positive urines**.

Effect	Frequentist results			Bayesian results		
	Estimate	95% CI	*T* (df), *p*-value	Estimate	95% CBI	*P* (O.R.<1|Data)
*d*-Amphetamine	0.987	0.966–1.008	−1.22 (323), *p* ≤ 0.223	0.987	0.943–1.023	0.772
Modafinil	1.007	0.993–1.022	1.03 (244), *p* ≤ 0.302	1.008	0.994–1.022	0.140
Modafinil + *d*-amphetamine	1.004	0.985–1.023	0.40 (184), *p* ≤ 0.692	1.012	0.946–1.074	0.330
Placebo	0.980	0.973–0.987	−6.09 (10), *p* ≤ 0.0001	0.979	0.958–0.998	0.985

*CBI, Bayesian 95% credible interval*.

### Side-effects, adverse events, compliance

Items most frequently endorsed on the weekly side-effects checklist are listed in Table 3. Participants in the *d*-amphetamine only group reported more symptoms throughout the study than the other groups; endorsing items suggestive of stimulant-like effects, e.g., increased energy, anxiety, and changes in sleep. Six study-related adverse events occurred: three involving cardiac-related symptoms (e.g., chest pain, change in EKG) in participants receiving combined modafinil and *d*-amphetamine group (*N* = 1), modafinil only (*N* = 1), and *d*-amphetamine only (*N* = 1). In two cases of reported chest pain, the study medication was discontinued and subjects were sent for cardiology evaluation at the nearby emergency clinic. Both subjects returned to the clinic within 3 days with no further symptoms. In the case of the EKG, non-specific ST-T wave changes at week 3 were reviewed by cardiology to rule out the possibility of new injury or ischemia. Study medication (modafinil + *d*-amphetamine) was discontinued. The subject returned to the clinic 1 week later, reporting no cardiovascular symptoms and showing improvement on repeat EKG. The other three events included pneumonia (modafinil), migraine (modafinil), and constipation (modafinil + *d*-amphetamine). All of these events were reviewed and approved by the IRB and Data Safety Monitoring Board. Medication adherence rates based on two measures: (1) self-reported days in which “all” study medications were taken; and (2) riboflavin-positive urines, were in the moderate range and not different across groups: combined modafinil and *d*-amphetamine group (65.8; 76.8%), modafinil only (64.5; 70%), *d*-amphetamine only (80.2; 67.1%), and placebo (73.2; 66.7%).

**Table 3:** **Items most frequently endorsed on the weekly side-effects checklist**.

Item	*d*-Amphetamine	Modafinil	Modafinil + *d*-amphetamine	Placebo	Total
Increase energy	22	10	5	9	46
Changes in sleep patterns	14	6	5	10	35
Anxiety or tension	10	9	6	8	33
Change in sexual performance or desire	16	5	6	6	33
Problems concentrating	12	4	7	7	30

## Discussion

This study found no evidence that the dual-agonist combination of modafinil and *d*-amphetamine had benefit over individual stimulant medications or placebo in the treatment of cocaine dependence. Participants receiving the medication combination showed a trend of increased cocaine use over time with a corresponding low Bayesian probability of benefit (33%). Relatively better cocaine outcomes were observed in the placebo and *d*-amphetamine only groups. The study medications were generally well-tolerated with few adverse effects, yet rates of medication adherence were less than optimal.

Of the many pharmacological strategies that have been investigated for cocaine dependence, those that “work” presumably via restoration of extracellular dopamine levels have shown efficacy for reducing drug use compared with placebo. Two such medications, each showing initial positive outcomes, dextroamphetamine (Grabowski et al., 2001, 2004) and modafinil (Dackis et al., 2005; Anderson et al., 2009), but each having different dopamine-enhancing mechanisms, were expected to produce more powerful treatment effects when given in combination. Our negative results, however, call into question the adequacy of this medication combination. The lower dose of each agent was combined in this preliminary investigation, leaving open the possibility that more robust actions and effects require higher doses. In general, data from previous studies indicate greater effectiveness of agonist medication when dosing at the high end of the recommended range, including 60 mg/day or higher of *d*-amphetamine (Grabowski et al., 2001, 2004). Higher doses of stimulant medications may be especially appropriate for patients with more severe stimulant dependence (Herin et al., 2010), as in the present sample. Whereas our pharmacological objective was to enhance dopamine; others have argued for using stimulant medications having broader actions on additional systems (e.g., 5-HT, NE) known to be involved in the neurochemical effects of cocaine (Rothman et al., 2001). Ideally being able to predict response to agonist treatment based on underlying biological tone would prove useful in medication matching (Elkashef and Vocci, 2003).

This study employed a longer duration of treatment (16 weeks), based on preliminary findings suggesting a delay in the onset of maximum treatment effects for *d*-amphetamine (Grabowski et al., 2001). In hindsight, it is clear that extending treatment should be accompanied by methods to enhance retention. High attrition, especially beyond week 12, was a major limitation of this preliminary study. Incentive-based strategies have been shown to increase visit attendance substantially (Businelle et al., 2009; Schmitz et al., 2010). Such strategies have also been effective for enhancing medication compliance (Schmitz et al., 2010) which was also less than optimal in the current study. To the extent that pill-taking burden in this study, i.e., three capsules daily, contributed to medication non-compliance, it is advisable for future medication trials to consider newer amphetamine formulations that have slower onset and longer-lasting efficacy (e.g., lisdexamfetamine: NCT00958282).

We opted to test this novel medication combination in a small number of subjects first to obtain evidence of efficacy and safety before designing a larger confirmatory study. Preliminary studies like this, however, have limitations, including unavoidable low statistical power and unreliable estimates. To address this uncertainty, we included Bayesian probability distributions that are not affected by sample size and can provide more quantitative conclusions regarding the probable magnitude of effects (Gurrin et al., 2000). In addition to “accepting” the null hypothesis based on conventional non-significant results (*p* < 0.05), Bayesian results informed us of only a 33% probability that treatment with combination of modafinil and *d*-amphetamine reduced cocaine-positive urines, given the current data. Thus, we can conclude with reasonable confidence that there is very little evidential support for conducting a larger study of this medication combination for treatment of cocaine dependence.

## Conflict of Interest Statement

The authors declare that the research was conducted in the absence of any commercial or financial relationships that could be construed as a potential conflict of interest.

## References

[B1] Anderson A. L. Reid M. S. Li S. H. Holmes T. Shemanski L. Slee A. Smith E. V. Kahn R. Chiang N. Vocci F. Ciraulo D. Dackis C. Roache J. D. Salloum I. M. Somoza E. Urschel H. C. III Elkashef A. M. (2009). Modafinil for the treatment of cocaine dependence. Drug Alcohol Depend. 104, 133–139 10.1016/j.drugalcdep.2009.04.015 19560290 PMC2818032

[B2] Bayarri M. J. Berger J. O. (2004). The interplay of bayesian and frequentist analysis. Stat. Sci. 19, 58–80 10.1214/088342304000000116

[B3] Businelle M. S. Rash C. J. Burke R. S. Parker J. D. (2009). Using vouchers to increase continuing care participation in veterans: does magnitude matter? Am. J. Addict. 18, 122–129 10.1080/10550490802545125 19283563

[B4] Carroll K. M. Fenton L. R. Ball S. A. Nich C. Frankforter T. L. Shi J. Rounsaville B. J. (2004). Efficacy of disulfiram and cognitive behavior therapy in cocaine-dependent outpatients: a randomized placebo-controlled trial. Arch. Gen. Psychiatry 61, 264–272 10.1001/archpsyc.61.3.264 14993114 PMC3675448

[B5] Dackis C. A. Kampman K. M. Lynch K. G. Pettinati H. M. O’Brien C. P. (2005). A double-blind, placebo-controlled trial of modafinil for cocaine dependence. Neuropsychopharmacology 30, 205–211 10.1038/sj.npp.1300701 15525998

[B6] Del Boca F. K. Kranzler H. R. Brown J. Korner P. F. (1996). Assessment of medication compliance in alcoholics through UV light detection of a riboflavin tracer. Alcohol. Clin. Exp. Res. 20, 1412–1417 10.1111/j.1530-0277.1996.tb01142.x 8947318

[B7] Elkashef A. Vocci F. (2003). Biological markers of cocaine addiction: implications for medications development. Addict. Biol. 8, 123–139 10.1080/1355621031000117356 12850771

[B8] Ferraro L. Tanganelli S. O’Connor W. T. Antonelli T. Rambert F. Fuxe K. (1996). The vigilance promoting drug modafinil increases dopamine release in the rat nucleus accumbens via the involvement of a local GABAergic mechanism. Eur. J. Pharmacol. 306, 33–39 10.1016/0014-2999(96)00182-3 8813612

[B9] Grabowski J. Rhoades H. Schmitz J. Stotts A. Daruzska L. A. Creson D. Moeller F. G. (2001). Dextroamphetamine for cocaine-dependence treatment: a double-blind randomized clinical trial. J. Clin. Psychopharmacol. 21, 522–526 10.1097/00004714-200110000-00010 11593078

[B10] Grabowski J. Rhoades H. Stotts A. Cowan K. Kopecky C. Dougherty A. Moeller F. G. Hassan S. Schmitz J. (2004). Agonist-like or antagonist-like treatment for cocaine dependence with methadone for heroin dependence: two double-blind randomized clinical trials. Neuropsychopharmacology 29, 969–981 10.1038/sj.npp.1300392 15039761

[B11] Grabowski J. Roache J. D. Schmitz J. M. Rhoades H. Creson D. Korszun A. (1997). Replacement medication for cocaine dependence: methylphenidate. J. Clin. Psychopharmacol. 17, 485–488 10.1097/00004714-199712000-00008 9408812

[B12] Green C. E. Moeller F. G. Schmitz J. M. Lucke J. F. Lane S. D. Swann A. C. Lasky R. E. Carbonari J. P. (2009). Evaluation of heterogeneity in pharmacotherapy trials for drug dependence: a Bayesian approach. Am. J. Drug Alcohol Abuse 35, 95–102 10.1080/00952990802647503 19322730 PMC4429522

[B13] Gurrin L. C. Kurinczuk J. J. Burton P. R. (2000). Bayesian statistics in medical research: an intuitive alternative to conventional data analysis. J. Eval. Clin. Pract. 6, 193–204 10.1046/j.1365-2753.2000.00216.x 10970013

[B14] Herin D. V. Rush C. R. Grabowski J. (2010). Agonist-like pharmacotherapy for stimulant dependence: preclinical, human laboratory, and clinical studies. Ann. N. Y. Acad. Sci. 1187, 76–100 10.1111/j.1749-6632.2009.05145.x 20201847

[B15] Longo M. Wickes W. Smout M. Harrison S. Cahill S. White J. M. (2010). Randomized controlled trial of dexamphetamine maintenance for the treatment of methamphetamine dependence. Addiction 105, 146–154 10.1111/j.1360-0443.2009.02717.x 19839966

[B16] Madras B. K. Xie Z. Lin Z. Jassen A. Panas H. Lynch L. Johnson R. Livni E. Spencer T. J. Bonab A. A. Miller G. M. Fischman A. J. (2006). Modafinil occupies dopamine and norepinephrine transporters in vivo and modulates the transporters and trace amine activity in vitro. J. Pharmacol. Exp. Ther. 319, 561–569 10.1124/jpet.106.105312 16885432

[B17] Margolin A. Kosten T. R. Avants S. K. Wilkins J. Ling W. Beckson M. Arndt I. O. Cornish J. Ascher J. A. Li S. H. Bridge P. (1995). A multicenter trial of bupropion for cocaine dependence in methadone-maintained patients. Drug Alcohol Depend. 40, 125–131 10.1016/0376-8716(95)01198-6 8745134

[B18] McLellan A. T. Kushner H. Metzger D. Peters R. Smith I. Grissom G. Pettinati H. Argeriou M. (1992). The fifth edition of the addiction severity index. J. Subst. Abuse Treat. 9, 199–213 10.1016/0740-5472(92)90062-S 1334156

[B19] Moeller F. G. Schmitz J. M. Herin D. Kjome K. L. (2008). Use of stimulants to treat cocaine and methamphetamine abuse. Curr. Psychiatry Rep. 10, 385–391 10.1007/s11920-008-0062-x 18803911 PMC9157609

[B20] Mooney M. E. Herin D. V. Schmitz J. M. Moukaddam N. Green C. E. Grabowski J. (2009). Effects of oral methamphetamine on cocaine use: a randomized, double-blind, placebo-controlled trial. Drug Alcohol Depend. 101, 34–41 10.1016/j.drugalcdep.2008.10.016 19058926 PMC2742691

[B21] Petrakis I. L. Carroll K. M. Nich C. Gordon L. T. McCance-Katz E. F. Frankforter T. Rounsaville B. J. (2000). Disulfiram treatment for cocaine dependence in methadone-maintained opioid addicts. Addiction 95, 219–228 10.1046/j.1360-0443.2000.9522198.x 10723850

[B22] Poling J. Oliveto A. Petry N. Sofuoglu M. Gonsai K. Gonzalez G. Martell B. Kosten T. R. (2006). Six-month trial of bupropion with contingency management for cocaine dependence in a methadone-maintained population. Arch. Gen. Psychiatry 63, 219–228 10.1001/archpsyc.63.2.219 16461866

[B23] Rothman R. B. Baumann M. H. Dersch C. M. Romero D. V. Rice K. C. Carroll F. I. Partilla J. S. (2001). Amphetamine-type central nervous system stimulants release norepinephrine more potently than they release dopamine and serotonin. Synapse 39, 32–41 10.1002/1098-2396(20010101)39:1<32::AID-SYN5>3.0.CO;2-3 11071707

[B24] Sayre S. L. Evans M. Hokanson P. S. Schmitz J. M. Stotts A. L. Averill P. Grabowski J. (2004). “Who gets in?” Recruitment and screening processes of outpatient substance abuse trials. Addict. Behav. 29, 389–398 10.1016/j.addbeh.2003.08.010 14732428

[B25] Schmitz J. M. Lindsay J. A. Stotts A. L. Green C. E. Moeller F. G. (2010). Contingency management and levodopa-carbidopa for cocaine treatment: a comparison of three behavioral targets. Exp. Clin. Psychopharmacol. 18, 238–244 10.1037/a0019195 20545388 PMC3164487

[B26] Schmitz J. M. Mooney M. E. Moeller F. G. Stotts A. L. Green C. Grabowski J. (2008). Levodopa pharmacotherapy for cocaine dependence: choosing the optimal behavioral therapy platform. Drug Alcohol Depend. 94, 142–150 10.1016/j.drugalcdep.2007.11.004 18164144 PMC2293271

[B27] Schmitz J. M. Stotts A. L. Rhoades H. M. Grabowski J. (2001). Naltrexone and relapse prevention treatment for cocaine-dependent patients. Addict. Behav. 26, 167–180 10.1016/S0306-4603(00)00098-8 11316375

[B28] Shearer J. (2008). The principles of agonist pharmacotherapy for psychostimulant dependence. Drug Alcohol Rev. 27, 301–308 10.1080/09595230801927372 18368612

[B29] Shearer J. Wodak A. van Beek I. Mattick R. P. Lewis J. (2003). Pilot randomized double blind placebo-controlled study of dexamphetamine for cocaine dependence. Addiction 98, 1137–1141 10.1046/j.1360-0443.2003.00447.x 12873248

[B30] Spitzer R. L. First M. B. (2005). Classification of psychiatric disorders. JAMA 294, 1898–1899; author reply 1899–1900. 10.1001/jama.294.15.1898 16234493

[B31] Stout R. L. Wirtz P. W. Carbonari J. P. Del Boca F. K. (1994). Ensuring balanced distribution of prognostic factors in treatment outcome research. J. Stud. Alcohol Suppl. 12, 70–75 7723001 10.15288/jsas.1994.s12.70

